# Editorial: Brain-Targeted Autoimmunity: Beyond Multiple Sclerosis

**DOI:** 10.3389/fimmu.2021.677577

**Published:** 2021-04-01

**Authors:** Serge Nataf, Stéphane Hunot, Guillaume Dorothée, Roland Liblau

**Affiliations:** ^1^ Univ Lyon, Université Claude Bernard Lyon 1, Inserm, Stem Cell and Brain Research Institute U1208, Bron, France; ^2^ Sorbonne Université, Institut du Cerveau ‐ Paris Brain Institute ‐ ICM, Inserm, CNRS, APHP, Hôpital de la Pitié Salpêtrière, Paris, France; ^3^ Sorbonne Université, Inserm, Centre de Recherche Saint-Antoine, CRSA, Immune System and Neuroinflammation Laboratory, Hôpital Saint-Antoine, Paris, France; ^4^ Infinity, Université Toulouse, CNRS, Inserm, UPS, Toulouse, France; ^5^ CHU Toulouse, Hôpital Purpan, Department of Immunology, Toulouse, France

**Keywords:** autoimmunity, central nervous system, autoantibodies, T-cell, neurodegeneration

## Introduction

There is mounting evidence indicating that brain-targeted autoimmunity develops in a large range of neurological conditions in which the involvement of the adaptive immune system was not previously envisioned. Beyond multiple sclerosis and a few central nervous system (CNS) disorders acknowledged to be autoimmune by nature, it now appears that multiple neurological conditions are accompanied by brain-targeted autoimmune processes. The eleven articles gathered in this Research Topic fall into four main research themes, which represent the likely focus of major investigative efforts in the near future: i) links between physiological and pathological brain-targeted autoimmunity, ii) the functional impact of post-lesional brain-targeted autoimmunity, iii) the role of brain-targeted autoimmunity in neurodevelopmental and neurodegenerative disorders, iv) the emergence of new clinical entities associated with brain-targeted autoimmunity. In this editorial, we first provide a brief summary of the main advances presented by these articles. We then discuss the concept of “brain-targeted autoimmunity” and how to refine it in order to improve our pathophysiological understanding of a growing array of CNS diseases.

## Unraveling the Links Between Physiological and Pathological Brain-Targeted Autoimmunity

In their article, Arevalo-Martin et al. show that in patients suffering from spinal cord injury, elevated levels of autoantibodies directed against CNS antigens can be identified as early as 5 days post-injury. These autoantibodies were determined to be naturally-occurring following their detection in the serum of healthy individuals as well. Along this line, Chantran et al. propose an original pathophysiological scheme in which naturally-occurring autoantibodies directed against the amyloid beta peptide Aβ1-40 may be responsible for the development of cerebral amyloid angiopathy complications. Finally, the data mining article by Nataf et al. points to a potential role of B-cells in shaping physiological autoimmune responses against neurodegeneration-associated proteins such as amyloid beta, alpha-synuclein and tau.

## Post-Lesional Brain-Targeted Autoimmunity

Owens et al. demonstrate that in patients suffering from intractable pediatric epilepsy, irrespective of etiology, brain-infiltrating T-cells are present in resected brain tissue samples. Thus, in both patients with immune-mediated epilepsy (more specifically Rasmussen encephalitis) and patients with epilepsy of non-immune origin, a robust immune response involving myeloid cells and T-cells is observed in the brain parenchyma. In their article, Javidi and Magnus review and discuss previous experimental findings assessing the kinetics and functional role of adaptive immune responses during the course of stroke and brain injury.

## Brain-Targeted Autoimmunity in Neurodevelopmental and Neurodegenerative Disorders

While Yshii et al. describe the pathophysiological mechanisms of paraneoplastic cerebellar degeneration and the resulting therapeutic implications that might be drawn from these experimental findings, Garretti et al. discuss recent data obtained in Parkinson’s disease patients and mouse models regarding the occurrence of autoreactive T-cells targeting neoepitopes of alpha-synuclein. Finally, Gata-Garcia and Diamond review the experimental and epidemiological evidence supporting the idea that maternal brain-reactive autoantibodies might impact fetal brain development and contribute to long-term behavioral and/or cognitive phenotypes, including autism spectrum disorders.

## Emergence of New Clinical Entities Associated With Brain-Targeted Autoimmunity

Three articles of this special issue relate to clinical, imaging and/or biological findings that are directly relevant to the practice of neurology and the diagnosis, therapeutic approach, and management of patients with autoimmune CNS disorders. Such pathologies include autoimmune glial fibrillary acidic protein astrocytopathy (Shan et al.) and autoimmune encephalitis (Lv et al.; Nóbrega et al.).

## Conclusion

Articles from this Research Topic highlight that the definition of “brain-targeted autoimmunity” requires further investigation and clarification. Obviously, demonstrating that autoantibodies and/or T-cells react against brain antigens under pathological conditions is not sufficient to formally conclude that such autoimmune processes play a significant pathophysiological role. This is particularly true when it comes to inferring that brain-targeted autoimmunity is a *primum movens*. Thus, animal models are critical to causally implicate such brain-targeted autoimmune processes in related disorders, and to decipher the underlying cellular and molecular mechanisms. With this framework, the paper by Yshii et al. offers a nice illustration of such experimental evidence in the context of paraneoplastic cerebellar degeneration. It becomes even more important to develop experimental approaches in animal models in light of new evidence that autoimmunity against CNS antigens also develops during the course of CNS disorders which are not primarily autoimmune, including prevalent and devastating conditions such as stroke and brain trauma (Javidi and Magnus), epilepsy (Owens et al.) and spinal cord injury (Arevalo-Martin et al.). Interestingly, at least in the context of stroke and spinal cord injury, CNS-targeted adaptive immune responses are found to develop early in the course of CNS injury and to likely stem from naturally-occurring autoantibodies and pre-existing self-reactive T cells. Such findings suggest that, while exerting physiological functions in the steady state, naturally-occurring adaptive immune responses might induce disease-specific effects which are somehow inhibited under normal conditions. In line with this view, the paper by Chantran et al. put forward an interesting pathophysiological scheme in which naturally-occurring autoantibodies directed against amyloid beta are proposed to serve protective functions during aging but can also contribute to complications of cerebral amyloid angiopathy in some individuals. Although the mechanisms underlying the development of naturally-occurring brain-targeted autoimmunity are still uncertain, the data mining analyses reported by Nataf et al. indicate that human B-cells physiologically express a large range of proteins involved in neurodegenerative processes and that these cells might be involved in the antigenic presentation of peptides derived from such proteins. On this basis, the authors posit that a main function of physiological brain-targeted autoimmunity is to prevent the accumulation of aggregated forms of amyloid beta, alpha-synuclein and/or tau proteins in the periphery. Although this assertion seems contradictory to the evidence of pathogenic autoimmune responses in patients with neurodegenerative conditions, Garretti et al. illustrate that pathogenic autoimmune T-cell responses against alpha-synuclein are indeed directed against neoepitopes, which occur under specific inflammatory conditions. Therefore, it is conceivable that protective and deleterious autoimmune responses to alpha-synuclein are directed against distinct epitopes and may actually co-exist in the same individual. This point is of particular importance as it suggests that harnessing the adaptive immune system for treating neurodegenerative disorders might require epitope-specific manipulations in order to achieve effectiveness and safety.

## Author Contributions

SN wrote the paper. RL, SH and GD provided corrections. All authors contributed to the article and approved the submitted version.

## Conflict of Interest

The authors declare that the research was conducted in the absence of any commercial or financial relationships that could be construed as a potential conflict of interest.

